# A genome-wide linkage scan for body mass index on Framingham Heart Study families

**DOI:** 10.1186/1471-2156-4-S1-S97

**Published:** 2003-12-31

**Authors:** Roxana Moslehi, Alisa M Goldstein, Michael Beerman, Lynn Goldin, Andrew W Bergen

**Affiliations:** 1 Genetic Epidemiology Branch, Division of Cancer Epidemiology and Genetics, National Cancer Institute, NIH, DHHS, Bethesda, Maryland, USA; 2 Core Genotyping Facility, Advanced Technology Center, National Cancer Institute, NIH, DHHS, Gaithersburg, Maryland, USA

## Abstract

**Background:**

Genome-wide scan data from a community-based sample was used to identify the genetic factors that affect body mass index (BMI). BMI was defined as weight (kg) over the square of height (m), where weight and height were obtained from the first measurement available between the ages of 40 and 50 years.

**Results:**

Significant familial correlations were observed in mother:father (spouse) relative pairs and in all relative pairs examined except parent:daughter pairs. Single-point sib-pair regression analysis provided nominal evidence for linkage (*p *< 0.05) of loci to BMI at 23 markers. Multi-point sib-pair regression analysis provided nominal evidence for linkage to BMI at 42 loci on 12 chromosomes. Empirical *p*-values showed results consistent with the multi-point results; all but three of the loci identified by multi-point analysis were also significant.

**Conclusion:**

The largest regions of nominally significant linkage were found on chromosomes 2, 3, and 11. The most significant evidence for linkage was obtained with markers D2S1788, D2S1356, D2S1352, D3S1744, and D11S912 from multi-point sib-pair single-trait regression analysis. Our results are in agreement with some of the recently published reports on BMI using various data sets including the Framingham Heart Study data.

## Background

Data from the ongoing NHLBI-supported Framingham Heart Study (FHS) of factors that contribute to cardiovascular disease (CVD) was made available to the Genetic Analysis Workshop 13 (GAW13). The FHS has collected physical exam and lifestyle information from a community-based sample that has identified major CVD risk factors, such as high blood pressure, cholesterol, smoking, and obesity . Obesity is a major risk factor for morbidity and mortality from many chronic diseases including CVD [1]. It is known that obesity-related traits such as BMI are influenced by genetic factors with heritability estimates of 40–90% [2]. Most studies have provided evidence for a polygenic etiology of common forms of obesity and BMI [2]. To identify the obesity and BMI loci, genome-wide linkage analyses have been performed in several populations including Europeans [3], Mexican Americans [4-6], Pima Indians [7,8], and African Americans [5,9]. In these studies, possible linkage (3 > LOD > 2) of obesity and obesity-related traits such as BMI and diabetes to markers on chromosomes 2, 3, 6, 7,11, 13, 17, and 20 were reported.

Recently two groups reported results of genome-wide linkage scans for BMI. Wu et al. found strong evidence for the presence of a quantitative trait locus (QTL) for BMI at 3q27 (marker D3S2427, LOD = 3.40, *p *= 0.03) in a meta-analysis of studies performed on individuals with Caucasian-, African-, and Mexican-American ethnic ancestry [5]. At least two other studies support linkage of BMI to this region. In a genome scan involving 507 nuclear families, Kissebah et al. reported the presence of a QTL locus on 3q27, which was linked to six traits including BMI [10]. Vionnet et al. also reported significant linkage with obesity-related diabetes to the same region of chromosome 3q [11]. Recent reports for linkage of BMI to other chromosomes includes a study by Deng et al. that identified a major QTL for BMI on 2q14 near the marker D2S347 (LOD = 4.04) in a Caucasian-American sample ascertained through a low-BMI proband [12]. One recent study on the FHS families reported linkage of BMI to chromosome 6q (marker D6S1009, LOD = 4.64) [13]. This study also reported some evidence of linkage to chromosome 11q14 markers, namely D11S4464 and D11S912 [13].

In the present study, we performed a genome scan using BMI and subjects from Framingham Cohorts 1 and 2. We chose to perform sib-pair analysis because the sib-pair method is an efficient way of screening for linked loci and is robust to generational differences in a trait such as BMI. Furthermore, we chose to limit the BMI measurements to a single time-point (ages 40–50) in an attempt to minimize the positive correlation of age on BMI.

## Methods

Subjects for this analysis were participants in the ongoing NHLBI-supported Framingham Heart Study (FHS), which included 5209 subjects in Cohort 1 who were recruited in 1948 and 5124 children of the original subjects and spouses of these children in Cohort 2 who were recruited in 1971. Selected data on these subjects were made available to GAW13. The available data included family histories on all FHS participants, phenotypic information including height and weight data, as well as results of genetic analysis of 401 polymorphic markers on chromosomes 1 to 22 in the largest families.

The data investigated for phenotypic assessment and familial correlation in this analysis were obtained from Cohort 1 and Cohort 2 and included the BMI calculated from the height and weight information provided by the FHS. The first recorded height and weight measurement between the ages of 40 and 50 years for each subject was used for the analysis. BMI was calculated by dividing weight in kilograms by the square of height in meters. The average BMI at ages 40 to 50 for individuals in Cohorts 1 and 2, 25.70 kg/m^2 ^and 26.54 kg/m^2^, respectively, differed significantly (*p *< 0.01, one-tailed t-test). We tested for significance of sex, cohort, and calendar year on BMI values across the entire data set of 2252 individuals. Sex was significant in all models while cohort was significant when included in models without calendar year. Calendar year was never significant (results not shown). Therefore sex and cohort were used as covariates in all linkage analyses. Our assumption was that sibs would be highly correlated for age at BMI and calendar year of BMI. We tested this assumption; this will be discussed later (see Discussion). Individuals without height and weight measurements between ages 40 to 50 years were removed from all analyses.

GAW13 phenotypic and genotypic data were imported into a Microsoft Access database and exported in appropriate files for familial correlation and linkage analysis in S.A.G.E. (Statistical Analysis for Genetic Epidemiology) 4.2 [14]. Familial correlations and the asymptotic standard errors were estimated using FCOR from S.A.G.E. 4.2. We used GENIBD from S.A.G.E 4.2 to generate identity-by-descent (IBD) sharing distributions of the GAW13 data. We applied single-marker as well as multiple-marker IBD analyses to generate IBD distributions for each pair of relatives at each marker. SIBPAL from S.A.G.E 4.2 was used to perform single regression of the mean corrected trait cross-product using IBD information with one marker being evaluated at a time. Furthermore, empirical *p*-values for all the markers were calculated using the SIBPAL program from S.A.G.E. 4.2. The covariates sex and cohort were included in the regression models.

## Results

The total number of individuals in the total FHS sample supplied to GAW13 is 4692, where 1702 have genotype information and 2252 have weight and height information. The total number of participants with BMI ≥ 30 (i.e., obese) is 248. Two hundred and forty-five individuals among the 1702 that were genotyped lacked data for BMI between the specified age range and were removed from all analyses. Eight individuals had BMI values that were 4 standard deviations above the mean (mean = 25.98). These eight outliers, all female with mean BMI of 47.5, were removed from all analyses.

Familial correlation analysis was done using S.A.G.E. 4.2 (FCOR). Table 1 provides the correlation coefficient and 95% confidence intervals for various relative pairs (sex-specific and non-sex-specific). The most significant familial correlation is seen among brother:brother pairs.

A genome-wide linkage analysis was performed using the GENIBD and SIBPAL programs of S.A.G.E 4.2. Through single-point linkage analysis, nominal evidence (*p *< 0.05) for linkage to BMI was found at 23 markers on 12 chromosomes (Table 2). Evidence for linkage to BMI at a *p*-value of less than 0.01 was found with markers D3S1764, D3S3053, D16S516, and D19S246 (Table 2). The strongest evidence for linkage using single-point analysis was found at D19S246 (*p *= 0.000051) on chromosome 19q.

Multi-point linkage analysis identified 42 markers on 12 chromosomes with *p*-values < 0.05. Empirical *p*-values for all these markers were calculated and are consistent with multi-point values (Table 3). Through multi-point analysis, evidence for linkage to BMI at a *p*-value of less than 0.01 was found with 15 markers (D1S552, D2S1788, D2S1356, D2S1352, D2S2739, D3S1764, D3S1744, D3S1763, D3S3053, D3S2427, D10S1435, D11S4464, D11S912, D11S2359, D19S246) (Table 3). The strongest evidence for linkage using multi-point analysis was found at D11S912 (*p *= 0.00009; *p*(emp) = 0.0003) on chromosome 11q (Table 3). The most significant results were with consecutive markers on chromosomes 2p (55.51–73.61 cM), 3q (152.62–188.29 cM), and 11q (123.0–147.77 cM) (Table 2).

**Figure 1:**
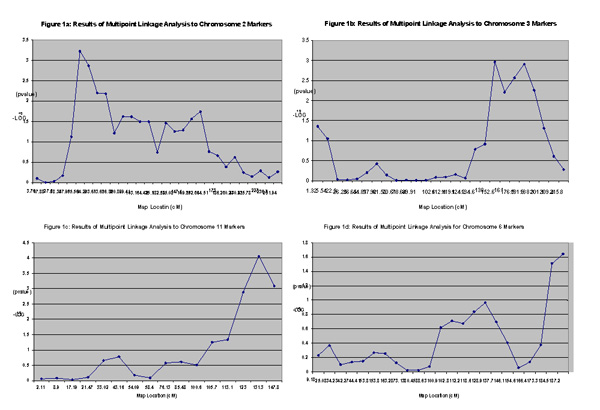
Results of multi-point linkage analysis to markers on (A) chromosome 2, (B) chromosome 3, (C) chromosome 11, and (D) chromosome 6

To further evaluate the linkage results, -Log_10_(*p*-value) was plotted for all markers for chromosomes 2, 3, and 11 that showed the strongest evidence for linkage (Figures 1a,1b,1c). -Log_10_(*p*-value) was also plotted for all markers on chromosome 6 for comparison with the findings of Atwood et al. [13] (Figure 1d).

## Discussion

The results of the familial correlation analysis on this data set provides evidence that significant familial correlations exist for BMI (Table 1) with all sibling pair and parent:son pair types tested, but that both genetic and environmental factors influence this correlation. The rank order of the point-estimate of familial correlation by relative pair type is brother:brother > brother:sister ~ sibling ~ mother:father > paternal:son ~ sister:sister. Brother:brother and brother:sister relative pairs are the only sex-specific relative pair type that have familial correlations for BMI with greater point-estimates than the correlation between mothers and fathers (i.e., spouses), however, brother:brother familial correlation is substantially greater than any other familial correlation. This may be due to the effect of same-sex environmental factors on BMI. When sibling pairs are evaluated for familial correlation without respect to sex, the point estimate of familial correlation for BMI is slightly greater than that of mother:father familial correlation.

In the present study, nominal evidence of linkage to BMI has been found with closely spaced markers on chromosomes 2, 3, and 11 (Table 3, Figure 1a,1b,1c). The results of multi-point analysis (Table 3) are consistent with the single-point analysis results (Table 2) on markers with *p*-values <0.01. The empirical *p*-values are also consistent with the *p*-values obtained from multi-point analysis (Table 3).

Because of the significant difference in BMI between Cohorts 1 and 2, which is consistent with secular upward trends in obesity over the last several decades, we tested the significance of calendar year of BMI in the 40 to 50 age range with respect to BMI values. Calendar year was not found to be an important factor and we expected age at BMI to be highly correlated among the sibs in our analysis with calendar year. In order to test our assumption that age at BMI in the narrow 40 to 50 age range did not play an important role, we repeated the analysis for the most significant chromosomes (2, 3, and 11) with age at BMI as a covariate. We found that including age at BMI as a covariate did not affect the linkage results (data not shown).

At least three other studies have suggested linkage of BMI to marker D3S2427 and/or other markers within that region of chromosome 3. Marker D3S2427, located on 3q27, was found to be strongly linked to BMI in the study by Wu et al. [5]. In the study by Kissebah et al., another marker on 3q27 at the map location of 190 cM, which is very close to the map location of D3S2427 at 188.29 cM, was strongly linked to BMI [10]. Another study reported significant linkage to the same region of chromosome 3 with diabetes or glucose intolerance in individuals below the age of 45 [11]. Since obesity and diabetes are often correlated with each other, some of the same loci may increase susceptibility to both diabetes and obesity.

Several groups have also reported evidence of linkage of BMI to markers on chromosome 2. Deng et al. found significant linkage of BMI with marker D2S347 on 2q14 at map location 131.51 cM [12]. Comuzzie et al. reported linkage of serum leptin levels to marker D2S1788 at 55.51 cM on chromosome 2p [15]. Since leptin levels in humans are believed to be correlated with BMI, Comuzzie et al. suggested that this region of chromosome 2 might contain an important human obesity gene [15]. Sequence variation at a biologically plausible positional candidate gene, the proopiomelanocortin locus, has subsequently been associated with normal variation in serum leptin levels [8]. Our results are in agreement with those of Comuzzie et al.; we have found evidence for the presence of a QTL on the short arm of chromosome 2 (2p14) at map locations 55.51 cM to 86.82 cM, which includes marker D2S1788. Marker D2S347 reported by Deng et al. was not typed in the FHS families.

Our results supporting nominal evidence of linkage to markers D11S4464, D11S912, and D11S2359 on chromosome 11 (123–147.77 cM) are in agreement with reports by several groups. Hanson et al. reported strong evidence of linkage of BMI to markers D11S4464 and D11S912 at 136.6–143.9 cM on chromosome 11q in Pima Indians [6]. Atwood et al. found suggestive evidence of linkage with markers on this chromosome between 130 cM and 170 cM in six different variance-component linkage analyses of BMI [13].

Our results also suggest possible linkage of BMI to markers D6S503 and D6S1027 on chromosome 6 (184.51–187.23 cM) (Table 3, Figure 1d). Duggirala et al. reported linkage of certain obesity-related traits such as insulin levels and leptin concentrations to chromosome 6q markers at 135–161 cM in nondiabetics from Mexican-American families [16]. This group also reported a LOD score of greater than 2.0 for linkage of these traits to marker D6S503, which is in agreement with our findings. More recently Atwood et al. reported very strong evidence for linkage of BMI to marker D6S1009 at 137.7 cM on chromosome 6q in FHS families [13]. The linkage region in our multi-point trace does not include the marker reported by Atwood et al., though the single-point linkage results for chromosome 6 (Table 2) include markers flanking D6S1009. The differences between our results and those of Atwood et al. could possibly reflect the methodological differences between our two studies. Atwood et al. performed their analysis using six different measurements of BMI taken over a span of 28 years from 1971 to 1988 [13]. We chose to perform our analysis using a single measurement between the ages of 40 and 50 for all subjects. In addition, Atwood et al. used a variance-components method for linkage analysis, which was different from the method used by our group.

## Conclusions

We report evidence for nominally significant linkage of BMI to three regions on chromosomes 2, 3, and 11 by performing multi-point sib-pair single-trait regression analysis and calculating empirical *p*-values for all markers using the Framingham Heart Study data.

## Figures and Tables

**Table 1:** Results of Correlation Analysis for BMI.

Ratio	BMI	95% CI	N pairs
Sex-specific			
Father:son	0.1327	0.023–0.242	425
Father:daughter	NS	NS	445
Mother:son	0.1446	0.0486–0.241	464
Mother:daughter	NS	NS	494
Brother:brother	**0.3306**^A^	**0.227–0.434**	430
Sister:brother	**0.2030**	**0.114–0.292**	861
Sister:sister	0.1255	0.026–0.225	487
Mother:father	0.1954	0.066–0.325	346
Non-sex specific			
Parent-child	0.0677	0.0146–0.1208	1828
Sibling	**0.2035**	**0.1390–0.2680**	1778

^A^Bold type indicates familial correlation values > mother:father familial correlation.

**Table 2:** Results of single-point linkage analysis at *p *< 0.05.

Chromosome	Probe	Marker	Map Location (cM)^A^	N Pairs	*p*-value
1	GATA109	D1S1728	109.04	1020	0.024063
1	GATA44E05	D1S1656	245.05	229	0.018998
2	GATA86E02	D2S1788	55.51	1074	0.031688
2	ATA4F03	D2S1356	64.29	1047	0.018078
2	GATA8F03	D2S1779	86.82	1061	0.039047
3	GATA4A10	D3S1764	152.62	525	0.008547
3	GATA92B06	D3S3053	181.87	523	0.001138
3	GATA22F11	D3S2427	188.29	517	0.014268
3	AFM059XA9	D3S1262	201.14	521	0.013133
6	GATA31	D6S474	118.64	1039	0.043069
6	GATA184A08	unknown	146.06	1053	0.047150
6	GATA81B01	D6S1277	173.31	995	0.026657
9	GATA21F05	D9S922	80.31	1026	0.034356
10	GATA88F09	D10S1435	4.32	1040	0.043675
11	GATA6B09	D11S1392	43.16	1026	0.020729
11	GATA64D03	D11S4464	123.0	1038	0.022781
12	ATA27A06	D12S1042	48.70	1007	0.016776
14	ATA19H08	D14S592	66.81	1003	0.046295
14	MFD190	D14S53	86.29	725	0.025172
16	350VD1	D16S516	100.39	1000	0.002877
18	GATA11A06	D18S542	41.24	1017	0.044304
19	GATA44F10	D19S591	9.84	1020	0.022472
19	MFD232	D19S246	78.08	950	0.000051

^A^Genetic map locations were obtained from the Marshfield Clinic website:

**Table 3:** Results of multi-point linkage analysis at *p *< 0.05.

Chromosome	GAW	Marker	Map Location (cM)^A^	N Pairs	*p*-value	Empirical (*p*-values)^B^
1	c1g4	D1S3669	37.05	1191	0.0095	0.01018
1	c1g5	D1S552	45.33	1191	0.0062	0.0034
1	c1g11	D1S1665	102.02	1191	0.0193	0.03616
1	c1g12	D1S1728	109.04	1191	0.0409	**0.05515**
2	c2g6	D2S1788	55.51	1198	0.0007	0.0006
2	c2g7	D2S1356	64.29	1198	0.0014	0.0004
2	c2g8	D2S1352	73.61	1198	0.0063	0.0004
2	c2g9	D2S2739	73.61	1198	0.0066	0.0059
2	c2g11	D2S1394	90.82	1198	0.0239	0.03112
2	c2g12	D2S1777	99.41	1198	0.0243	0.02948
2	c2g13	D2S1790	103.16	1198	0.0321	0.02094
2	c2g14	D2S2972	114.42	1198	0.032	0.04966
2	c2g16	D2S1328	132.58	1198	0.0349	0.04485
2	c2g19	D2S1326	149.89	1198	0.0274	0.02124
2	c2g20	D2S1399	152.04	1198	0.0181	0.03957
3	c3g1	D3S4559	1.32	1191	0.0445	0.04413
3	c3g19	D3S1764	152.62	1191	0.0011	0.0043
3	c3g20	D3S1744	161.04	1191	0.0062	0.0009
3	c3g21	D3S1763	176.54	1191	0.0027	0.0035
3	c3g22	D3S3053	181.87	1191	0.0013	0.0015
3	c3g23	D3S2427	188.29	1191	0.0056	0.0059
3	c3g24	D3S1262	201.14	1191	0.049	**0.09426**
5	c5g23	D5S1471	172.13	1196	0.0266	0.0341
5	c5g24	D5S1456	174.8	1196	0.047	**0.0721**
6	c6g22	D6S503	184.51	1193	0.0308	0.04273
6	c6g23	D6S1027	187.23	1193	0.0227	0.03528
7	c7g14	D7S1799	113.92	1193	0.015	0.03686
8	c8g13	Unknown	110.2	1195	0.0375	0.03671
10	c10g1	D10S1435	4.32	1195	0.0094	0.0026
11	c11g13	D11S1998	113.13	1195	0.0456	**0.07096**
11	c11g14	D11S4464	123	1195	0.0013	0.0023
11	c11g15	D11S912	131.26	1195	0.00009	0.0003
11	c11g16	D11S2359	147.77	1195	0.0008	0.0012
15	c15g1	D15S822	12.3	1196	0.0304	0.0102
15	c15g2	D15S817	4.78	1196	0.0158	0.0207
15	c15g3	D15S165	20.24	1196	0.0161	0.02733
15	c15g4	Unknown	31.46	1196	0.0454	0.01775
16	c16g3	D16S764	29.97	1195	0.0427	0.03495
16	c16g4	D16S403	43.89	1195	0.0385	0.02171
16	c16g10	D16S2624	87.62	1195	0.0247	0.02007
16	c16g11	D16S516	100.39	1195	0.0031	0.01558
19	c19g9	D19S246	78.08	1193	0.0027	0.0055

^A^Genetic map locations were obtained from the Marshfield Clinic website: ^B^Values in bold are above 0.05.
